# Biomarker‑driven phase Ib clinical trial of OPB‑111077 in acute myeloid leukemia

**DOI:** 10.3892/mi.2022.32

**Published:** 2022-02-22

**Authors:** Joaquín Martínez-López, Pau Montesinos, Nieves López-Muñoz, Rosa Ayala, Pilar Martínez-Sánchez, Julian Gorrochategui, José Luis Rojas-Rudilla, Daniel Primo, Juan-Miguel Bergua-Burgues, María Calbacho, Evelyn Acuña-Cruz, José Antonio Pérez-Simón, Adolfo De La Fuente, Jaime Pérez De Oteyza, Rebeca Rodriguez-Veiga, José Sánchez Pina, Blanca Boluda, Isabel Cano, María Liz Paciello Coronel, Juan Ballesteros

**Affiliations:** 1 Department of Hematology, 12 de Octubre Hospital, Instituto de Investigación Hospital 12 de Octubre (i+12), Complutense University, H12O-CNIO Clinical Research Unit, CIBERONC, 28041 Madrid, Spain; 2 Department of Hematology and Hemotherapy, La Fe University and Polytechnic Hospital, 46026 Valencia, Spain; 3 Department of Hematology, 12 de Octubre Hospital, 28041 Madrid, Spain; 4 Bioinformatics, Vivia Biotech, 28760 Madrid, Spain; 5 VP Science, Vivia Biotech, 28760 Madrid, Spain; 6 Department of Hematology, San Pedro de Alcántara Hospital, 10003 Cáceres, Spain; 7 Department of Hematology, Virgen del Rocio University Hospital, Institute of Biomedicine of Sevilla (IBIS/CSIC, CIBERONC), University of Sevilla, 41013 Sevilla, Spain; 8 Department of Hematology, MD Anderson Cancer Center, 28033 Madrid, Spain; 9 Department of Hematology, HM Sanchinarro University Hospital, School of Medicine, University CEU San Pablo, 28050 Madrid, Spain; 10 Vivia Biotech, 28760 Madrid, Spain

**Keywords:** relapsed/refractory acute myeloid leukemia, STAT3, OPB-111077, *ex vivo* sensitivity test, maximum tolerated dose

## Abstract

OPB-111077 is a novel, highly specific oral signal transducer and activator of transcription 3 inhibitor that has exhibited good efficacy against solid and blood cancers, including acute myeloid leukemia (AML), in preclinical models. In the present study, a phase 1b, two-stage, 3+3 dose-escalation clinical trial [dose level (DL)1 of 200 mg/day and DL2 of 250 mg/day on a once daily dose schedule in 28-day cycles] was conducted to assess the maximum tolerated dose (MTD), safety profile and the preliminary antitumor activity of OPB-111077 in patients with high-risk AML. A preliminary preclinical analysis evaluated the anti-proliferative activity of OPB-111077 in 19 patients with AML with a Vivia Biotech *ex vivo* PharmaFlow precision medicine test. A total of 12 patients were ultimately enrolled in the trial: 5 patients (42%) were treated with DL1, and 7 (58%) were escalated to DL2 of OPB-111077. Dose-limiting toxicities were not observed and the MTD was not reached. In addition, the most frequently reported treatment-emergent adverse events were nausea, vomiting and fatigue. Finally, clinical activity (overall response) was observed in 3 patients (25%). On the whole, the present study demonstrates that OPB-111077 exhibits a good safety and tolerability profile and an acceptable clinical response in patients with high-risk AML. A biomarker-driven design is useful for selecting the study population upfront.

## Introduction

Acute myeloid leukemia (AML) is a heterogeneous clonal hematopoietic progenitor cell disorder characterized by immature myeloid cell proliferation and bone marrow failure, exhibiting a spectrum of morphological, immunophenotypic, cytogenetic and molecular characteristics (1).

Moreover, AML is an aggressive disease with a poor prognosis (2,3). In addition, >50% of patients with AML are not candidates for intensive chemotherapy therapy due to their age, performance status and/or associated comorbidities (4). Although the long-term overall survival (OS) rates of patients <65 years of age have significantly improved over the past years owing to improved supportive care and an increased use of allogeneic hematopoietic stem cell transplantation (alloHSCT), the prognosis for the elderly AML population is still poor, with a 5-year OS rate of <10% (5).

Furthermore, two-thirds of patients with AML who achieve a complete remission (CR) will relapse within the following 18 months (6), and regrettably, there are no safe and effective curative treatments, apart from alloHSCT, which is a rather aggressive therapeutic modality with high treatment-related morbidity and mortality (5). Therefore, given the significant incidence of relapsed AML and the frequent toxicities associated with standard intensive chemotherapy, an optimal treatment strategy for this population remains unsatisfactory and has yet to be established (4,7). In addition, although several new drugs for the treatment of AML, particularly for elderly patients, have been approved in recent years, such as the FMS-like tyrosine kinase 3 inhibitors, venetoclax, glasdegib or Vyxeos, the medical needs of patients with relapsed or refractory (RR) AML continue to be unmet (1,2,8,9).

Signal transducer and activator of transcription (STAT) is a seven-member family group of latent cytoplasmic transcription factors that are involved in hematopoietic cytokine receptor signaling pathways that mediate several biological processes, such as cell proliferation, differentiation, survival and immune response, by transferring signals from cell-surface cytokines and growth factor receptors to the cell nucleus and subsequently by regulating the transcription of target genes (10). The persistent and aberrant activation of specific STAT factors, particularly STAT3, often results in the growth and survival of tumor cells and, consequently, in the development of a wide range of cancers (11). STAT3 is the STAT family member most strongly associated with tumorigenesis. There are two main mechanisms through which STAT3 promotes tumorigenesis: By acting as a nuclear transcription factor (12) and as a regulator of oxidative phosphorylation (OXPHOS) via interaction with components of the electron transport chain (13).

STAT3 is constitutively activated in leukemic cells from patients with AML. It is now clear that the activation of STAT3 contributes to the development and resistance of AML (10). Furthermore, the assessment of bone marrow samples from patients with AML has demonstrated that the activation of STAT3 is significantly associated with a reduced OS and progression-free survival (PFS) (14).

It has been demonstrated that the blockade of aberrant STAT3 signaling induces tumor cell apoptosis and inhibits tumor growth, confirming its critical role in the molecular pathogenesis of several tumors. It has also been identified as a potential target for the discovery and development of novel anticancer drugs (10,15).

OPB-111077 is a novel orally bioavailable low-molecular-weight compound discovered and developed by Otsuka Pharmaceutical Co., Ltd. as an orally active antitumor agent for the treatment of various types of cancer. In preclinical analyses, it has been shown to be a potent and highly specific STAT3 inhibitor with a good efficacy and safety profile, supporting the initiation of early clinical investigation in humans (16). In fact, a first in-human study of OPB-111077 demonstrated that it could be administered safely, and its pharmacokinetic profile was acceptable for further clinical development (16). Mechanistic analyses have demonstrated that OPB-111077 significantly inhibits the STAT3 activation pathway, with antitumor effects against a wide range of human solid and blood tumor cell lines. Furthermore, OPB-111077 has been shown to exhibit efficacy against several solid and blood cancers both *in vitro* and *in vivo* (16,17).

Although in a phase I study, the activity of OPB-111077 in a range of solid tumors was limited, this drug exhibited clinical activity in one subject with diffuse large B-cell lymphoma (16), and hence, it could be more efficient in tumor cells with a high proliferative index, such as AML.

Biomarker-based treatment selection is a popular topic in oncology. However, few successful biomarkers have been discovered thus far, with the majority of these being molecular, such as tyrosine kinase inhibitors in chronic myeloid leukemia (18).

Previous studies have analyzed the association between *ex vivo* drug testing and clinical outcomes in adult patients with AML. Functional *ex vivo* assays that predict a patient's clinical response to anticancer drugs for guiding cancer treatment have long been a goal, but few have yet proven to be reliable (19,20).

The present study conducted a phase Ib dose-escalation and biomarker-driven study to assess the safety and efficacy profiles of OPB-111077 in patients with RR AML. In order to identify and select the subpopulation most sensitive to the study drug and optimize disease management, a precision medicine, personalized, *ex vivo* test was first performed that evaluated the pharmacological activity of OPB-111077 directly in individual patient bone marrow samples.

## Patients and methods

### Ethics approval

The present study was approved by the Research Ethics Committee of Hospital Universitario 12 de Octubre, Madrid, Spain, and was conducted according to all the local regulatory requirements, as well as in accordance with the Declaration of Helsinki. Informed consent was provided by all the study participants. This trial was registered at www.clinicaltrials.gov as # NCT03197714.

### Study population

Patients were eligible for the study if they met the following inclusion criteria: A diagnosis of RR non-M3-AML, newly diagnosed non-M3-AML not eligible for or willing to undergo intensive induction chemotherapy, and the highest sensitivity (>70% of the samples analyzed) in the bone marrow analysis of the OPB-111077 *ex vivo* sensitivity test. The other selection criteria are presented in Table SI. The following patient characteristics were collected: Age, weight, height, sex, Eastern Cooperative Oncology Group (ECOG) performance status, blast infiltration, FMS-like tyrosine kinase (FLT), nucleophosmin 1 (NPM1), French-American-British (FAB classification), the presence of concomitant disease, refractory AML and the number of relapses.

### Study design and treatment

This phase 1b, open-label, non-randomized, dose-escalation clinical trial comprised two stages. The first dose-escalation stage aimed to characterize the safety, tolerability and maximum tolerated dose (MTD) of OPB-111077 in patients with high-risk AML. Subsequently, following the determination of the MTD, an expansion stage further evaluated the safety and preliminary antitumor activity of OPB-111077 in the study population.

OPB-111077 was administered orally on a once daily dose schedule in 28-day cycles until intolerable toxicity or disease progression, with two dosing schemes as follows: A starting dose or dose level (DL)1 of 200 mg/day and a DL2 of 250 mg/day. A 3+3 dose-escalation schedule based on the dose-limiting toxicity (DLT) assessment following the first dose of OPB-111077 was implemented.

Patients were enrolled in the study between September 7, 2017 and March 31, 2020 at three Spanish sites: Hospital La Fe (Valencia), Hospital 12 de Octubre (Madrid), and Hospital San Pedro de Alcántara (Cáceres). Patients fulfilling the study selection criteria were included in the trial after evaluating their anti-proliferative activity to OPB-111077 with a Vivia Biotech *ex vivo* PharmaFlow precision medicine (PM) test (Vivia Biotech, S.L.) (21). This tool is a cell-based multicolor screening flow cytometry platform that evaluates the pharmacological activity of drug treatments on individual patient bone marrow samples, assessing the patient's cell sensitivity or resistance to a specific drug. Its methodology has been previously described in detail (22). The Vybrant^®^ CFDA SE Cell Tracer lit (Thermo Fisher Scientific, Inc.) was used to distinguish between proliferating and non-proliferating cells, and StemSpan™ Serum-Free Expansion Medium II (SFEM II; StemCell Technologies, Inc.) supplemented with StemSpan™ CC100 (StemCell Technologies, Inc.) and autologous plasma was used as the culture medium for proliferation *ex vivo* assays in both the preliminary preclinical phase, where the approved drug, decitabine, was also used as an anti-proliferative control, and later in the clinical trial. The leukemic cells were identified using a gating strategy based on forward scatter and/or side scatter and the expression of different surface markers. The response effect was measured by counting the number of live leukemic cells remaining following exposure to increasing concentrations of OPB-111077 in both the proliferating and non-proliferating fractions based on carboxyfluorescein diacetate (CFDA) expression. Dose response curves for the drug were measured for each proliferative subset based on the CFDA peak signal. A criterion to consider the results valid was set based on the culture behavior of tumor cells. Thus, tumor cells must be viable in culture (net difference with preincubation basal measure) and >40% confluent in control wells without the drug. In addition, the ratio of non-induced apoptosis could not be increased by >60%.

Data analysis for the estimation of the drug effect on pathological cells from bone marrow samples was carried out using a population modeling approach and a non-linear mixed effect regression analysis using NONMEM software version 7.2 (version VII, ICON Development Solutions). By this methodology, dose-response curves from all samples were calculated and processed simultaneously. Residual errors and interindividual variability were calculated to determine the population standard profile for the drug. The normalized value of the area under the dose-response curve (PERCENT_AUC) was used as the optimal activity marker that was derived from the estimated individual model parameters. Patients whose *ex vivo* results to OPB-111077 fell within the highest 30% (range, >70th percentile of the OPB-111077 profile) were classified as sensitive and enrolled in the study. In total, 26 out of 47 patients were initially discarded due to acceptance criteria of the 70th percentile (Fig. S1).

Optimal culture conditions were typically observed at 72 h; thus, the results were preferably evaluated at this incubation time. If an insufficient number of proliferative cells was counted or a high uncertainty was associated with the result estimations, the results were then evaluated at longer time periods of 96 or 120 h. Only single values with an acceptable range 95% confidence interval (CI) <40% were considered in any case.

### Safety assessments

The MTD level was defined as the maximum dose level below the maximum administered dose at which less than one-third of the patients experienced DLT. The study patients (between a minimum of 3 and a maximum of 12 patients) began on level 1, and they were assessed weekly during the first 28 days following the first dose of OPB-111077.

DLT was defined as one of the following toxicities occurring during the DLT assessment window and was considered by the investigator to be related to study treatment: Any grade ≥3 or 4 non-hematological toxicity or any unexpected non-tolerable grade II adverse event possibly related to the treatment regimen that requires a delay beyond 1 week until recovery.

The tolerability and safety of OPB-111077 assessment was assessed by recording the incidence of treatment-emergent adverse events (TEAEs) and by grading them according to the Common Terminology Criteria for Adverse Events (CTCAE) Version 4.03(23).

### Efficacy assessments

Bone marrow aspiration was performed on the 1st day of each cycle until the end of treatment (EOT). Following bone marrow aspiration, the clinical response was assessed with the overall response rate (ORR), which was defined as the percentage of patients who reached CR, morphological complete remission with incomplete blood count recovery (CrCRi) or partial remission (PR) (24). In the case of CR or CrCRi after cycle 3, bone marrow aspiration was performed every 3 months.

The EOT visit took place within 14 days after the final administration of the study drug or at the time of discontinuation from the trial. Patients discontinued the study if they experienced intolerable toxicity, suffered disease progression, withdrew their consent, or did not benefit from the trial therapy in the opinion of the investigator.

PFS was defined as the time from the date of the informed consent form to the date of progression or death (from any cause), whichever occurred first. OS was defined as the time from the date of the informed consent form to the date of death due to any cause.

### Statistical analysis

Exploratory and descriptive methods were used to describe all the study variables. Continuous variables are summarized as the mean, median, standard deviation and interquartile range, and categorical variables are presented as absolute and relative distributions of frequencies. Baseline categorical characteristics for enrolled and excluded patients due to screening failure were compared using the Chi-squared test (Table SII). The associations between ORR and the half maximal effective concentration (EC50) and the area under the curve (AUC), as determined using the Vivia Biotech *ex vivo* sensitivity test, were evaluated with an unpaired t-test. PFS and OS time-to-event analyses were performed using the Kaplan-Meier method; no comparisons were made for time-to-event outcomes and, therefore, no P-values are provided.

All analyses were performed using the SPSS Statistics software package, version 22.0 (IBM Corporation).

## Results

**Figure 1:**
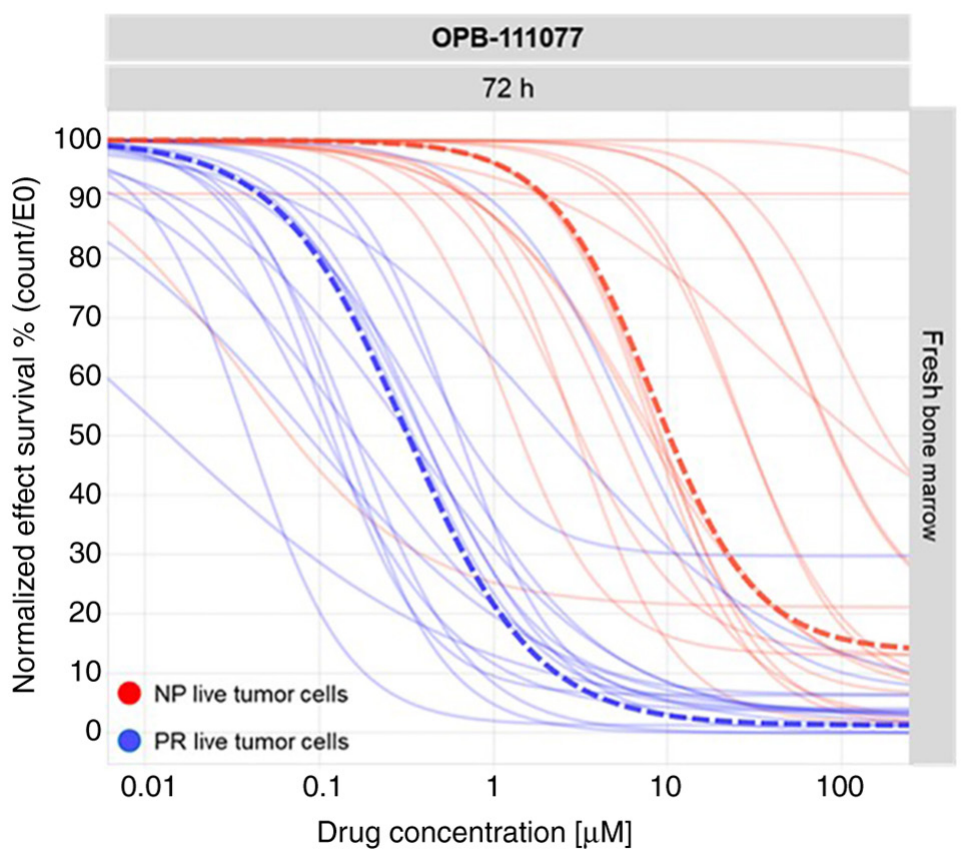
Preclinical overlapped dose-response curves of OPB111077 monotherapy. Cumulated results for fresh samples showing the *ex vivo* pharmacological profile of OPB-111077 at 72 h. Red samples represent the resistant non proliferating live tumor cells, and blue lines display the sensitive proliferating live tumor cells. The highlighted dotted lines represent the median of both sensitive proliferating (blue) and resistant non-proliferating (red) patients. NP, non proliferating; PR, proliferating.

To analyze the mechanisms of action of the compound, in a preliminary preclinical phase, a total of 19 patients with AML were analyzed at the Vivia Biotech laboratories in a proliferation assay. This was the starting point to further expand the number of samples reaching statistical significance and converging in population models in order to achieve a better characterization of OPB-111077. As shown in Fig. 1, OPB-111077 exerted anti-proliferative rather than cytotoxic activity, as it exerted a more prominent effect on proliferating cells than on the population of non-proliferative cells. A comparison between a reference anti-proliferative approved drug in AML, decitabine, was performed. Population dose response curves of the proliferating cells were generated using both the novel OPB-111077 compound and decitabine. The pharmacological profiles revealed a high interpatient variability in the patient samples incubated with OPB-111077 and in those incubated with decitabine (Figs. S2 and S3), suggesting the need for a precision medicine (PM) test to select the best patient candidates. The overlapping population curves of the proliferating cells showed similar activity of OPB-111077 vs. decitabine.

Once the pharmacodynamic model of OPB-111077 in the AML patient samples was established, a phase Ib investigator-sponsored trial using this assay as a selection criterion was launched. A total of 47 patients with RR AML were screened, and 12 were ultimately enrolled in the study between September 7, 2017 and March 31, 2020 at three Spanish sites (Fig. S1): Hospital La Fe (Valencia), Hospital 12 de Octubre (Madrid), and Hospital San Pedro de Alcántara (Cáceres). In total, 26 patients were excluded using the personalized medicine sensitivity test, as their results were below the primary acceptance criteria of the 70th percentile.

**Figure 2:**
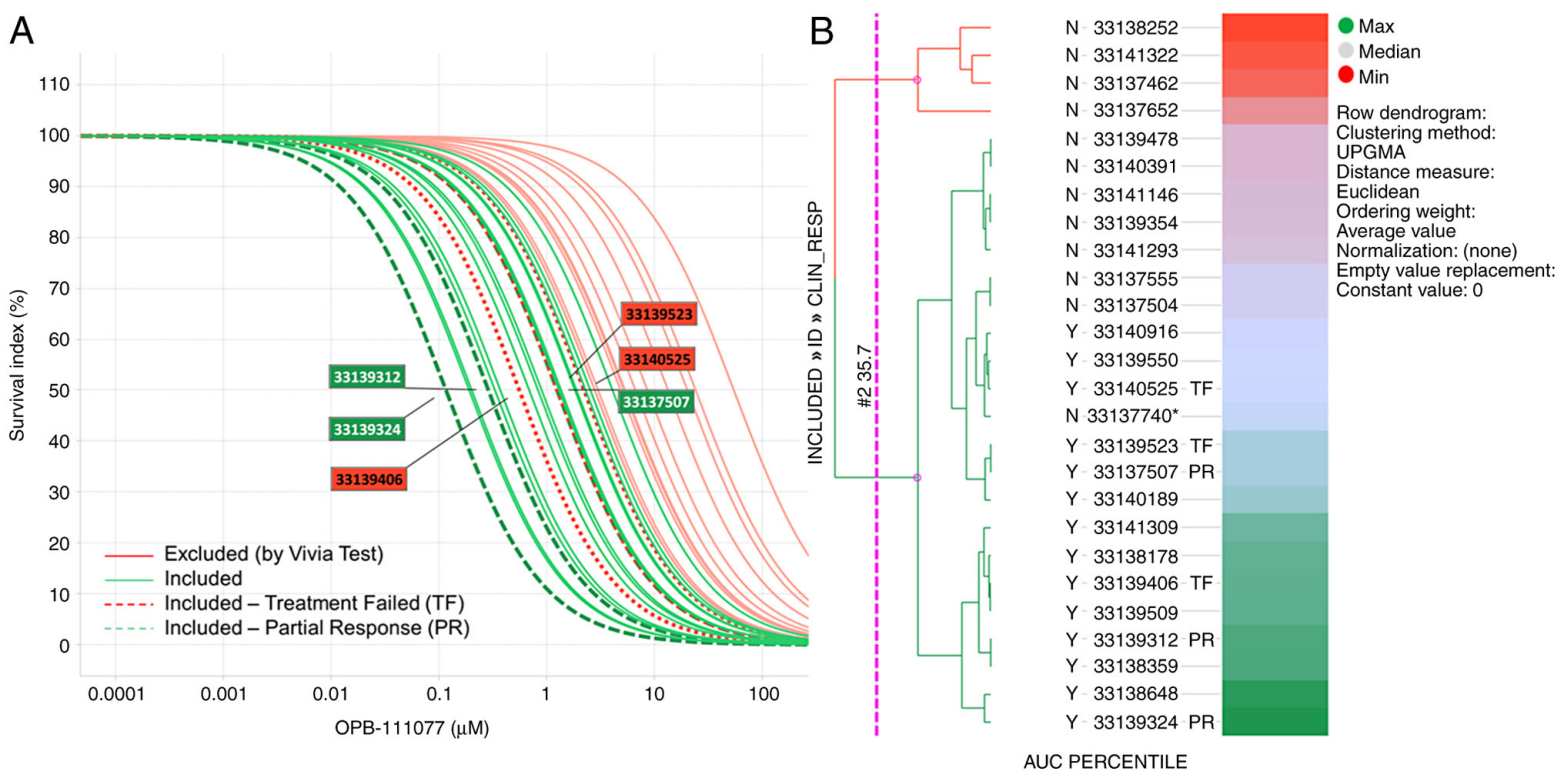
Pharmacological profile at 72 h of patients with acute myeloid leukemia included in the clinical trial. (A) Dose response curves of OBP-111077 in patient samples, highlighting sensitive (green) and resistant (red) treated patients. Dotted green lines shows the *ex vivo* sensitive patients who had partial response later, and the dotted red lines reflects the *ex vivo* less sensitive patients who had treatment failure. (B) Heatmap with the AUC percentile stratification. The greener the block, the more sensitive predictive model.

Dose response curves of OPB-111077 in bone marrow samples from the screened subjects, highlighting those sensitive and resistant treated patients, are displayed in Fig. 2A. A stratification based on the percentile AUC and represented in a heatmap was performed to aid in the selection of patients to be included in this phase Ib clinical trial (Fig. 2B). Few samples crossed the sensitive (green) vs. resistant (red) threshold. These samples near the threshold may have slightly shifted their activity from 72 h shown to 96 h or 120 h (data not shown), which were also measured and could serve to decide on patient inclusion.

The patient demographics and baseline clinical characteristics are summarized in Table I. The median age was 76 years, and 91.7% were male. No differences were observed in the patient screening failure, except in the frequency of the NPM1 mutation (Table SII). A total of 5 (42%) patients with AML were refractory; the median (range) of relapse was 2 (1-6) (Table I). In addition, 5 (42%) patients were treated with the first level dose (DL1) of OPB-111077 (200 mg), while 7 patients (58%) were escalated the second dose level (DL2) of 250 mg. The median total doses administered were 17,000 mg and 8,250 mg for DL1 and DL2, respectively. The study treatment dose was only reduced in 1 patient treated with DL2.

### Safety MTD

Dose-limiting toxicity was not observed in any of the patients treated with either DL1 (200 mg) or DL2 (250 mg); hence, the MTD was not reached.

### Safety assessments

The most frequently reported serious adverse events (SAEs) in the study population, ranging from grade 3 (G3) to grade 5 (G5), were febrile neutropenia, pneumonia and respiratory tract infection (Table II).

Moreover, seven TEAEs were reported in 3 patients, all with grades 1 or 2: One patient treated with DL1 experienced vomiting (G2); a second patient treated with DL2 had extrasystoles (G2); and a third patient treated with DL2 reported anorexia, diarrhea, epigastric discomfort, nausea and vomiting, all with G1. Only extrasystoles (G2) were regarded as a severe TEAEs.

All enrolled patients (n=12) discontinued the study treatment (Fig. S1). In total, 6 patients (50%) did so due to disease progression, and 3 (25%) did so as a result of adverse events [respiratory failure (G5), respiratory infection (G5), and extrasystoles (G2)]. Furthermore, 2 patients died during the treatment period due to disease progression and respiratory infection.

### Efficacy

Only 6 patients (50%) were evaluable for clinical efficacy, assessed as the ORR. A total of 6 patients (50%) were excluded from the clinical efficacy assessment as they either did not have a bone marrow aspiration or they had no information about cycle 2. Among the evaluable patients, 3 (25%) patients achieved PR, whereas the other 3 (25%) patients presented with treatment failure (TF) as the optimal response. ORR was therefore observed in 3 (25%) patients, with a 95% CI of 0.5-49.5%.

The biomarker AUC and EC50 values differed according to the clinical response. Patients with PR as the optimal response presented higher mean AUC values (80.94%) than those observed in patients with TF (59.91%), with a mean difference (95% CI) of 21.033 (-8.361-50.428). Likewise, and as shown in Fig. 2A, the median EC50 was lower in patients with PR as the best response (0.45 µM) than in patients with TF (1.28 µM), with a mean difference (95% CI) of 0.831 (-0.563-2.226). However, none of the observed differences reached statistical significance (P>0.05) (Table III).

Finally, the estimated median PFS and OS were 57 days (95% CI, 37-77) and 95 days (95% CI, 27-163), respectively, as shown in Figs. S4 and S5.

## Discussion

The present phase I dose-escalation trial was performed to assess the safety, tolerability and efficacy of OPB-111077 in patients with RR AML treated with doses ranging from 200-250 mg/day for 4 weeks.

In the current trial, no DLTs were observed, and therefore, the MTD (primary study endpoint) was not reached, confirming the good safety profile of OPB-111077. This good safety and tolerability profile has also been reported in previously published studies with OPB-111077 (16,17). Likewise, the most frequently reported TEAEs were nausea, vomiting and fatigue.

Although the clinical activity (i.e., an ORR of 25%) may be considered modest (25,26), it was much higher than the response observed in the aforementioned published phase I studies with OPB-111077 (i.e., an ORR of 1/145) (16,17). It is also an even higher response rate compared to other new drugs with different mechanisms of action, such as MDM2 antagonist RO6839921. Uy *et al* (27) reported a response rate of 7.7% in their phase 1 study. The same occurred in the phase 1 study on CWP232291, in which Lee *et al* (28) described a low number of responses. However, it should be noted that the patients included in the present clinical trial had a very poor prognosis; they were elderly (many of them >70 years of age), a difficult-to-treat population (29), and the majority were refractory to standard therapy (30). Tolcher *et al* (16) reported clinical activity (durable PR) in only one subject, with diffuse large B-cell lymphoma, from a population of 18 patients with unselected and mostly solid tumors, while in the study conducted by Yoo *et al* (17), no patients with hepatocellular carcinoma achieved complete or partial responses with OPB-111077. A plausible explanation for this finding is that, unlike other phase I trials, in the present study, the population was selected based on a biomarker that enabled the upfront identification and enrollment of those AML patients with the highest sensitivity to the study drug, discarding those hypothetically resistant ones and thus minimizing the likelihood of treatment failures. This is supported by the differences in both the AUC and EC50 values that were found between patients achieving PR or TF as the optimal responses. However, those differences did not meet the statistical significance criteria, probably due to the small sample size. Other research groups have also implemented this *ex vivo* personalized medicine sensitivity test in the AML population to improve prognostic risk stratification, tailor treatments, and minimize drug resistance. As in the current analysis based on the expression of a biomarker, other researchers have found strong correlations between the *ex vivo* sensitivity test and the clinical response to chemotherapy in AML patients in their respective studies (31,32).

One of the mechanisms through which STAT3 promotes oncogenesis is through the activation of OXPHOS (16). Of note, OXPHOS has been reported to be involved as a mechanism of resistance to chemotherapy in AML (33). Therefore, the use of drugs targeting OXPHOS may be an appropriate therapeutic approach for the treatment of refractory and relapsed AML (34,35). Other drugs have been proposed to function through the OXPHOS of leukemic cells, such as IACS-010759(36) and ME-344(37) (4). However, in contrast to OPB-111077, phase 1 studies of the use of these drugs in relapsed/refractory acute myeloid leukemia have not yet been conducted.

As demonstrated in the present study, drugs such as decitabine, similar to OPB-111077, exert an anti-proliferative effect on tumor cells. Therefore, the combination of both can increase anti-tumor activity. In this regard, the authors of an ongoing trial evaluating the combination of OPB-111077 with decitabine and venetoclax for the treatment of AML have suggested that the combination of OPB-111077 and venetoclax reduces tumor cell proliferation and increases apoptosis rates to a greater extent than exposure to any single study drug (38). Notably, the effects obtained with the combination were even more pronounced in AML cells that were genetically engineered to increase OXPHOS (38). Pollyea *et al* (39) also demonstrate that the combination of venetoclax and a hypomethylating agent such as azacitidine can eradicate leukemic cells by disrupting energy metabolism through suppression of OXPHOS. This is in line with the similar activity and weak toxicity found in the preliminary preclinical study we performed, which may suggest a similar clinical profile; thus, their use in combination could increase the chance of achieving an overall response.

Certain limitations of the present study are the small number of patients included, although this is due to of the nature of a phase I clinical trial and the strategy used for patient selection. The employment of an *ex vivo* test for selection could hinder patient treatment in this aggressive disease.

In conclusion, OPB-111077 as a monotherapy has exhibited a good safety and tolerability profile in patients with RR AML. Additionally, some clinical response was found compared to previous studies performed with the same study drug (16,17). The innovative biomarker-driven design used in the present study to select the patient population upfront based on their sensitivity to the study drug may partly explain these improved results over previous studies. This innovative phase IB biomarker selection design may help to lower the high attrition rate of new drugs.

## Supplementary Material

Patient enrollment flow chart. ST, sensitivity test (<70% of analyzed samples); DF, diagnosis failure (without histological evidence of relapsed or refractory acute myeloid leukemia); LE, life expectancy ≤3 months; SI, systemic antineoplastic therapy within 14 days of study treatment; IC, insufficient cellularity; OR, other reason (death caused by COVID-19 infection).

Population curves for OPB-111077 and decitabine. O.F.V^*^, objective function; parameters typical and random (variability and residual error) are shown together with the corresponding relative standard error calculated as the ratio between the standard error provided by NONMEM and the estimate. Estimates of inter-patient variability (IPV) are expressed as the coefficient of variation (%).

Overlapped populational curves of OPB-111077 and decitabine.

Cumulative progression-free survival. Kaplan-Meier survival curve. Median PFS (95% CI), 57.000 (36.631-77.369).

Overall survival. Kaplan-Meier survival curve. Median OS (95% CI), 95.000 (26.545-163.455).

Study selection criteria.

Patient characteristics.

## Figures and Tables

**Table I:** Clinical and demographic characteristics.

Characteristic	Value
Age, years, median (range)	76.0 (72.0-79.0)
Weight, kg, median (range)	69.0 (64.8-79.3)
Height, cm, median (range)	165.5 (160.0-169.5)
Sex, n (%)	
Female	1 (8.3)
Male	11 (91.7)
ECOG, n (%)	
0	5 (41.7)
1	6 (50.0)
Unknown	1 (8.3)
Blast Infiltration, %, median (range)	62.0 (47.0-71.0)
FLT-3 ITD, n (%)	
Not mutated	8 (66.7)
Mutated	2 (16.7)
Unknown	2 (16.7)
NPM1, n (%)	
Not mutated	4 (33.3)
Mutated	2 (16.7)
Unknown	6 (50.0)
FAB, n (%)	
M0	1 (12.5)
M1	3 (37.5)
M2	1 (12.5)
M4	1 (12.5)
M4 eos	1 (12.5)
M5	1 (12.5)
Concomitant disease, n (%)	
Yes	12 (100.0)
Refractory AML, n (%)	5 (41.7)
Relapses, median (range)	2 (1-6)

ECOG, Eastern Cooperative Oncology Group; FLT, FMS-like tyrosine kinase; NPM1, nucleophosmin 1; FAB: French-American-British classification.

**Table II:** Serious adverse events per subject.

			Grade									
			2		3		4		5		Total	
Dose level	SOC	PT	n	%	n	%	n	%	n	%	n	%
Level 1: 200 mg daily (n=5)	Blood and lymphatic system disorders	Febrile neutropenia	0	0.0	1	20.0	0	0.0	0	0.0	1	20.0
	Gastrointestinal disorders	Colitis	0	0.0	1	20.0	0	0.0	0	0.0	1	20.0
	Infections and infestations	Pneumonia	0	0.0	2	40.0	0	0.0	0	0.0	2	40.0
		Respiratory syncytial virus	0	0.0	0	0.0	0	0.0	1	20.0	1	20.0
		infection	0	0.0	1	20.0	0	0.0	0	0.0	1	20.0
		Respiratory tract infection	0	0.0	1	20.0	0	0.0	0	0.0	1	20.0
		Skin infection	0	0.0	1	20.0	0	0.0	0	0.0	1	20.0
		Soft tissue infection										
	Respiratory, thoracic and mediastinal disorders	Dyspnea	0	0.0	1	20.0	0	0.0	0	0.0	1	20.0
		Pulmonary hemorrhage	0	0.0	0	0	1	20.0	0	0.0	1	20.0
		Respiratory failure	0	0.0	0	0	0	0.0	1	20.0	1	20.0
Level 2: 250 mg daily (n=7)	Blood and lymphatic system disorder	Febrile neutropenia	0	0.0	2	28.6	0	0.0	0	0.0	2	28.6
	Cardiac disorders	Extrasystoles	1	14.3	0	0.0	0	0.0	0	0.0	1	14.3
	Infections and infestations	Pneumonia	0	0.0	0	0.0	0	0.0	1	14.3	1	14.3
		Respiratory tract infection	0	0.0	1	14.3	0	0.0	0	0.0	1	14.3
		Sepsis	0	0.0	1	14.3	0	0.0	0	0.0	1	14.3
		Septic shock	0	0.0	0	0.0	0	0.0	1	14.3	1	14.3
		Tonsillitis	0	0.0	1	14.3	0	0.0	0	0.0	1	14.3
	Injury, poisoning and procedural complications	Medication error	1	14.3	0	0.0	0	0.0	0	0.0	1	14.3
	Renal and urinary disorders	Acute kidney injury	1	14.3	0	0.0	0	0.0	0	0.0	1	14.3
Total (n=12)	Blood and lymphatic system disorders	Febrile neutropenia	0	0.0	3	25.0	0	0.0	0	0.0	3	25.0
	Cardiac disorders	Extrasystoles	1	8.3	0	0.0	0	0.0	0	0.0	1	8.3
	Gastrointestinal disorders	Colitis	0	0.0	1	8.3	0	0.0	0	0.0	1	8.3
	Infections and infestations	Pneumonia	0	0.0	2	16.7	0	0.0	1	8.3	3	25.0
		Respiratory syncytial virus infection	0	0.0	0	0.0	0	0.0	1	8.3	1	8.3
		Respiratory tract infection	0	0.0	2	16.7	0	0.0	0	0.0	2	16.7
		Sepsis	0	0.0	1	8.3	0	0.0	0	0.0	1	8.3
		Septic shock	0	0.0	0	0.0	0	0.0	1	8.3	1	8.3
		Skin infection	0	0.0	1	8.3	0	0.0	0	0.0	1	8.3
		Soft tissue infection	0	0.0	1	8.3	0	0.0	0	0.0	1	8.3
		Tonsillitis	0	0.0	1	8.3	0	0.0	0	0.0	1	8.3
	Injury, posioning and procedural complications	Medical error	1	8.3	0	0.0	0	0.0	0	0.0	1	8.3
	Renal and urinary disorders	Acute kidney injury	1	8.3	0	0.0	0	0.0	0	0.0	1	8.3
	Respiratory, thoracic and mediastinal disorders	Dyspnoea	0	0.0	1	8.3	0	0.0	0	0.0	1	8.3
		Pulmonary haemorrhage	0	0.0	0	0.0	1	8.3	0	0.0	1	8.3
		Respiratory failure	0	0.0	0	0.0	0	0.0	1	8.3	1	8.3

SOC, system organ class; PT, preferred term.

**Table III:** Clinical response according to biomarker AUC and EC50 in the sensitivity test.

Association between the optimal response and the mean AUC					
Optimal response	No. of patients	Mean AUC	SD AUC	Mean difference (95% CI)	P-value^a^
PR	3	80.943	12.584	21.033 (-8.361-50.428)	0.118
TF	3	59.910	13.338		
Association between the optimal response and the EC50 in the sensitivity test					
Best response	No. of patients	Mean EC50	SD EC50	Mean difference (95% CI)	P-value^a^
PR	3	0.453	0.445	0.831 (-0.563-2.226)	0.173
TF	3	1.285	0.748		

^a^Data were analyzed using a Student's t-test, PR, partial remission; TF, treatment failure; SD, standard deviation; AUC, area under the curve; EC50, half maximal effective concentration; CI, confidence interval.

## Data Availability

The datasets used and/or analyzed during the current study are available from the corresponding author on reasonable request.
