# Supplementary material for: Biomarker‑driven phase Ib clinical trial of OPB‑111077 in acute myeloid leukemia
Source: Med Int (Lond). 2022 Feb 22;2(2):7. doi: 10.3892/mi.2022.32 (PMC11208994; doi:10.3892/mi.2022.32)
Supplement: Patient enrollment flow chart. ST, sensitivity test (<70% of analyzed samples); DF, diagnosis failure (without histological evidence of relapsed or refractory acute myeloid leukemia); LE, life expectancy ≤3 months; SI, systemic antineoplastic therapy within 14 days of study treatment; IC, insufficie [file Supplementary_Data.pdf]

Figure S1. Patient enrollment flow chart. ST, sensitivity test (<70% of analyzed samples); DF, diagnosis failure (without histological evidence of relapsed or refractory acute myeloid leukemia); LE, life expectancy ≤3 months; SI, systemic antineoplastic therapy within 14 days of study treatment; IC, insufficient cellularity; OR, other reason (death caused by COVID-19 infection).

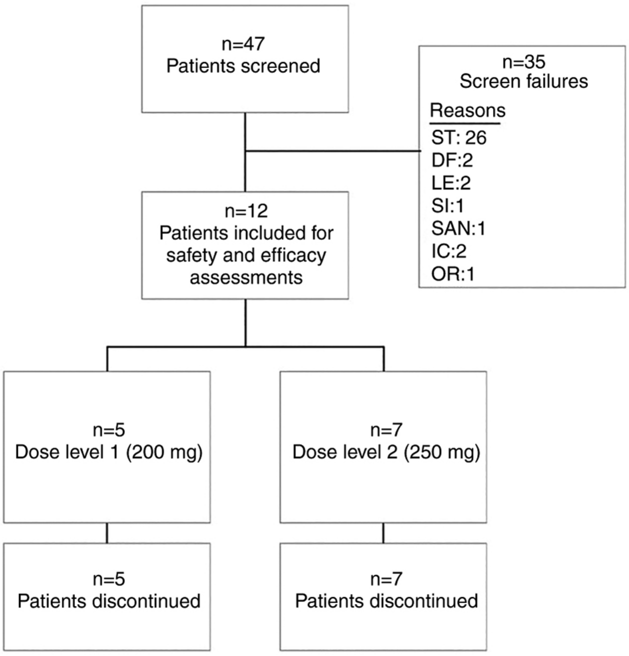

Figure S2. Population curves for OPB-111077 and decitabine. O.F.V*, objective function; parameters typical and random (variability and residual error) are shown together with the corresponding relative standard error calculated as the ratio between the standard error provided by NONMEM and the estimate. Estimates of inter-patient variability (IPV) are expressed as the coefficient of variation (%).

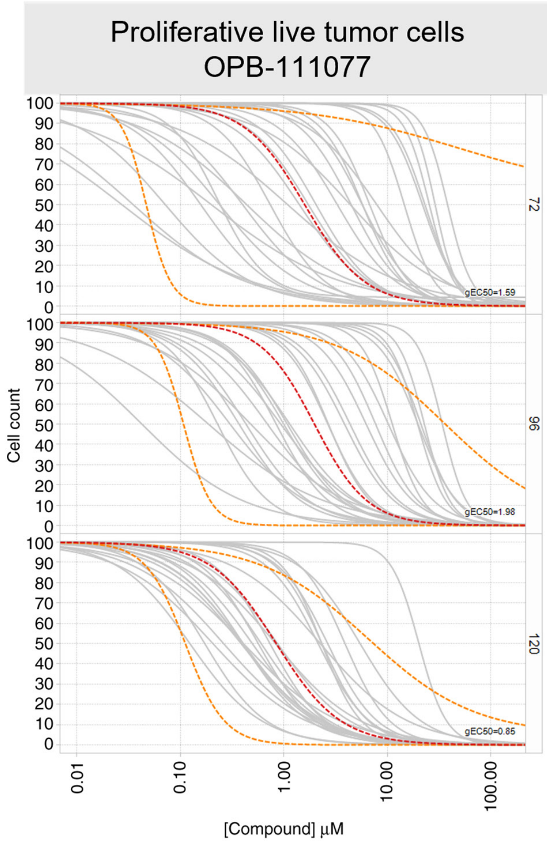


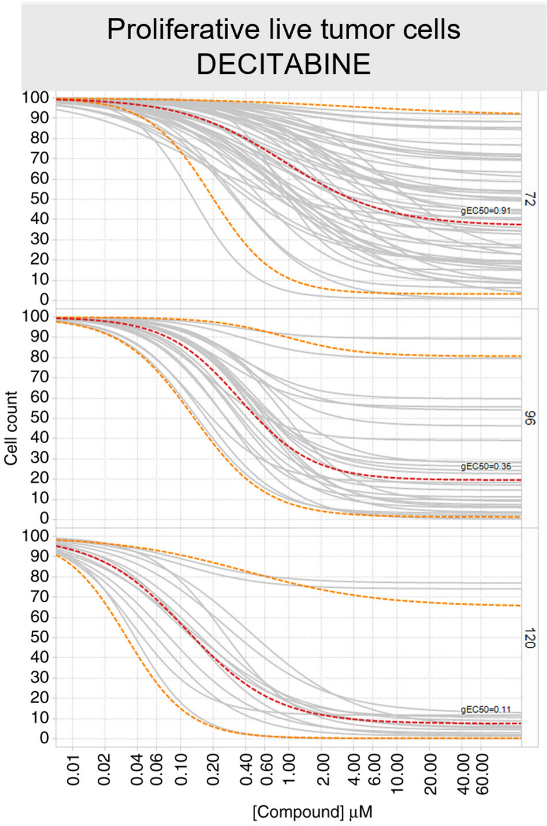


| | PD00202 | | | | | |
|---|---|---|---|---|---|---|
| | OPB-111077 | | | | | |
| | 72 | | 96 | | 120 | |
| | Values | SE(%) | Values | SE(%) | Values | SE(%) |
| N | 27 | | 26 | | 23 | |
| O.F.V* | 200.7 | | 127.3 | | 90.9 | |
| E0 | 1767 | 30.43 | 3285 | 70.68 | 5296 | 37.52 |
| Emax | 1.00 | 0.01 | 1.00 | 6.28 | 1.00 | 0.00 |
| EC50 | 1.5512 | 94.88 | 1.9921 | 73.28 | 0.8427 | 64.91 |
| GAMMA | 1.49 | 23.06 | 1.68 | 31.41 | 1.39 | 14.78 |
| Additive error | 0.4288 | 11.21 | 0.4097 | 21.81 | 0.4239 | 9.01 |
| E0.IPV (%) | 121.8140 | 39.77 | 101.2280 | 98.54 | 77.5356 | 55.69 |
| Emax.IPV (%) | 3460.1900 | 390.09 | 950.2370 | 99.60 | 3510.9300 | 2730.66 |
| EC50.IPV (%) | 444.6880 | 61.28 | 310.9270 | 145.32 | 153.5090 | 59.66 |
| GAMMA.IPV (%) | 30.0369 | 53.23 | 16.9655 | 211.87 | 9.2393 | 52.65 |
| A.Error.IPV (%) | 40.3896 | 25.63 | 16.4237 | 148.66 | 12.2684 | 57.21 |

| | PD00038 | | | | | |
|---|---|---|---|---|---|---|
| | DECITABINE | | | | | |
| | 72 | | 96 | | 120 | |
| | Values | SE(%) | Values | SE(%) | Values | SE(%) |
| N | 52 | | 25 | | 14 | |
| O.F.V* | -460.5 | | -159.7 | | -154.9 | |
| E0 | 1482 | 19.38 | 2397 | 25.07 | 3830 | 49.15 |
| Emax | 0.63 | 11.99 | 0.80 | 0.04 | 0.93 | 13.90 |
| EC50 | 0.9108 | 20.88 | 0.3480 | 58.73 | 0.1134 | 19.76 |
| GAMMA | 0.93 | 11.52 | 1.29 | 20.55 | 1.05 | 15.30 |
| Additive error | 0.2216 | 12.54 | 0.2814 | 16.02 | 0.2124 | 26.41 |
| E0.IPV (%) | 109.8290 | 25.44 | 110.7500 | 53.17 | 82.3147 | 47.19 |
| Emax.IPV (%) | 301.4090 | 22.49 | 300.9720 | 410.68 | 379.6860 | 45.35 |
| EC50.IPV (%) | 88.3504 | 39.18 | 35.0894 | 83.64 | 61.9014 | 84.75 |
| GAMMA.IPV (%) | 8.2206 | 65.49 | 0.0000 | 60.53 | 5.1747 | 96421358.41 |
| A.Error.IPV (%) | 36.9259 | 22.57 | 42.0754 | 39.35 | 32.9384 | 53.27 |

Figure S3. Overlapped populational curves of OPB-111077 and decitabine.

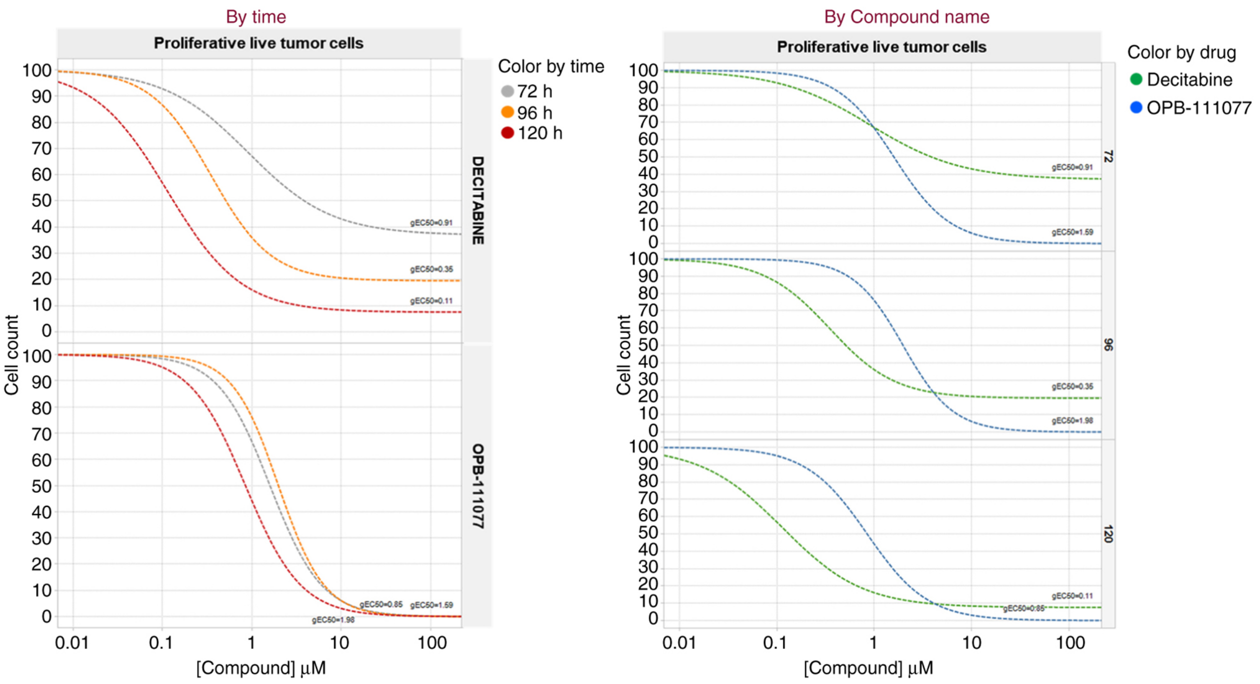

Figure S4. Cumulative progression-free survival. Kaplan-Meier survival curve. Median PFS (95% CI), 57.000 (36.631-77.369).

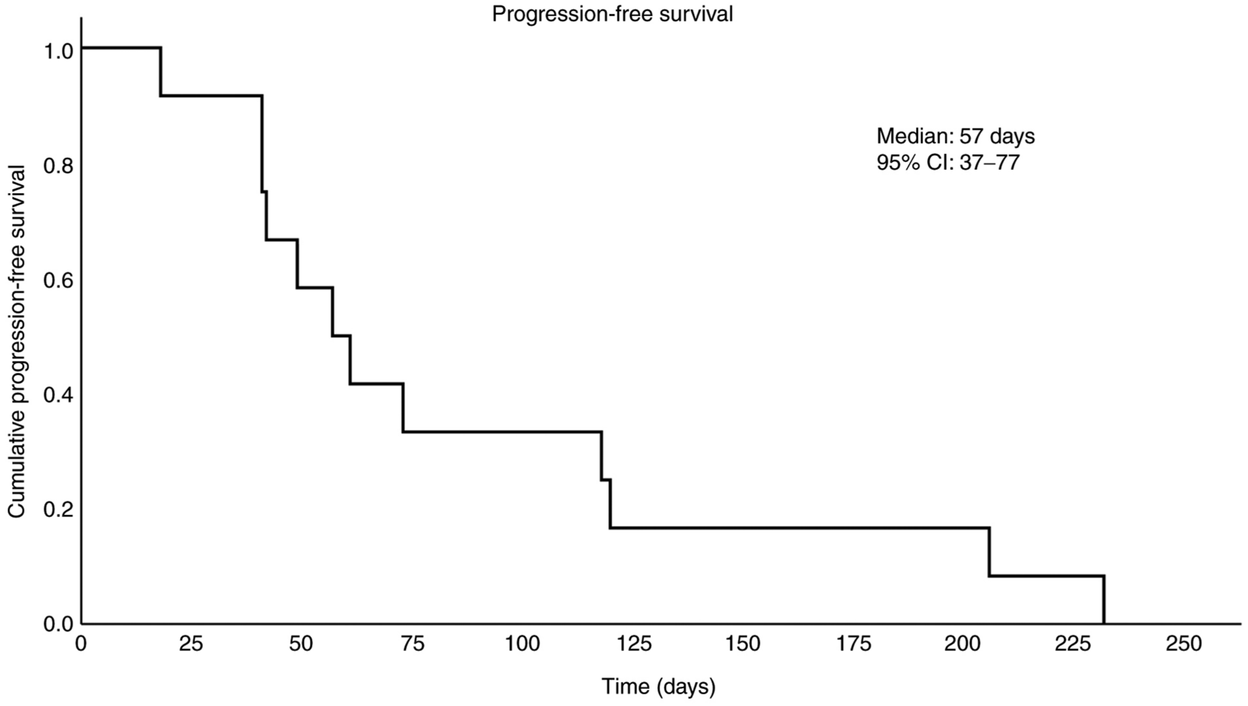

Figure S5. Overall survival. Kaplan-Meier survival curve. Median OS (95% CI), 95.000 (26.545-163.455).

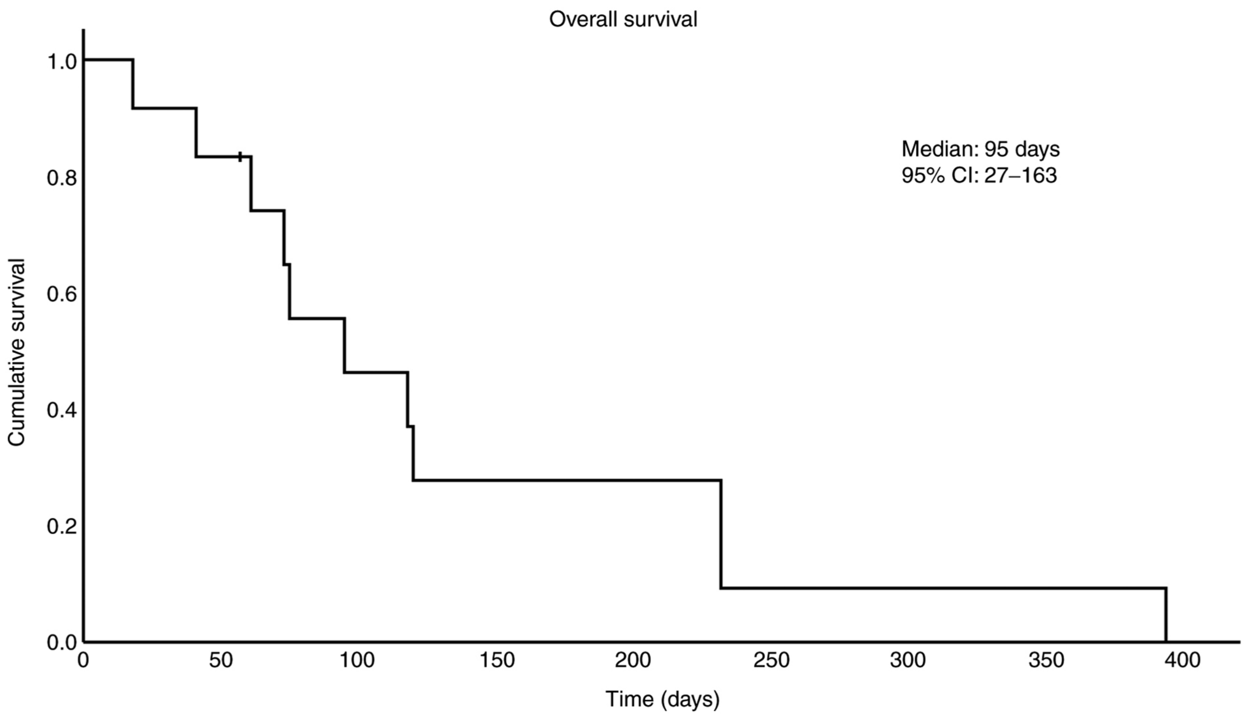

Table SI. Study selection criteria.

| | |
|---|---|
| Inclusion criteria | •Diagnosis of relapsed or refractory non M3-AML or newly diagnosed non M3-AML patients who are not eligible for or unwilling to undergo intensive induction chemotherapy.<br>•ECOG PS <2.<br>•Life expectancy >3 months.<br>•Adequate renal, hepatic and cardiac functions (defined as: Serum creatinine <2X ULN or CrCl >40 ml/min; bilirubin <2X ULN; ALT and AST <2.5X ULN).<br>•LVEF >50%.<br>•NYHA CHF: Class II or better.<br>•A highest sensitivity (higher 70% of the samples analyzed) in the bone marrow analysis of the OPB-111077 *ex vivo* sensitivity test. |
| Exclusion criteria | •Medical history of other malignancies.<br>•Diagnosis of M3/APL leukemia.<br>•Any systemic antineoplastic therapy received within 14 days before study entry.<br>•Any investigational drug within 28 days before the first dose of study treatment. |

AML, acute myeloid leukemia; M3/APL, acute promyelocytic leukemia; ECOG, Eastern Cooperative Oncology Group; PS, performance status; ULN, upper limit of normal; CrCl, creatinine clearance; ALT, alanine aminotransferase; AST, aspartate aminotransferase; LVEF, left ventricular ejection fraction; NYHA, New York Heart Association; CHF, congestive heart failure.

Table SII. Patient characteristics.

| Parameter | Total (n=36) | Enrolled patients (n=12) | Screening failure (n=24) | P-value[a] |
|---|---|---|---|---|
| Age, years, median (range) | 67 (26-80) | 76 (72-79) | 63.4 (26-80) | |
| Sex n (%) | | | | 0.42 |
| Male | 25 (69.4) | 11 (91.7) | 14 (58.3) | |
| Female | 11 (30.6) | 1 (8.3) | 10 (41.7) | |
| ECOG PS | | | | 0.57 |
| 0 | 14 (38.8) | 5 (41.7) | 9 (37.5) | |
| 1 | 18 (50.0) | 6 (50.0) | 12 (50.0) | |
| 2 | 2 (5.5) | 0 (0) | 2 (8.3) | |
| Unknown | 2 (5.5) | 1 (8.3) | 1 (4.2) | |
| FLT3-ITD, n (%) | | | | 0.297 |
| Not mutated | 29 (80.5) | 8 (66.7) | 21 (87.5) | |
| Mutated | 3 (8.3) | 2 (16.7) | 1 (4.2) | |
| Unknown | 4 (11.1) | 2 (16.7) | 2 (8.3) | |
| NPM1, n (%) | | | | 0.002 |
| Not mutated | 26 (72.2) | 4 (33.3) | 22 (91.7) | |
| Mutated | 2 (5.5) | 2 (16.7) | 0 (0) | |
| Unknown | 8 (2.2) | 2 (16.7) | 2 (8.3) | |
| Number of previous lines, n (%) | | | | 0.079 |
| 1 | 9 (25) | 1 (8.3) | 8 (33.3) | |
| 2 | 15 (41.7) | 7 (58.3) | 8 (33.3) | |
| 3 | 8 (22.2) | 2 (16.7) | 6 (25.0) | |
| 4 | 1 (2.8) | 0 (0) | 1 (4.2) | |
| 5 | 1 (2.8) | 1 (8.3) | 0 (0) | |
| 6 | 1 (2.8) | 1 (8.3) | 0 (0) | |
| Unknown | 1 (2.8) | 0 (0) | 1 (4.2) | |

[a]Data were analyzed using the Chi-square test. ECOG PS, Eastern Cooperative Oncology Group Performance Status; FLT3-ITD, FMS-like tyrosine kinase 3, internal tandem duplication; NPM, nucleophosmin.
